# Acute effects of unilateral and bilateral conditioning activity on countermovement jump, linear speed, and muscle stiffness: A randomized crossover study

**DOI:** 10.1371/journal.pone.0292999

**Published:** 2023-10-13

**Authors:** Piotr Biel, Mateusz Zubik, Aleksandra Filip-Stachnik, Paulina Ewertowska, Michał Krzysztofik

**Affiliations:** 1 Department of Sport and Physical Education, AGH University of Science and Technology, Krakow, Poland; 2 Institute of Sport Sciences, The Jerzy Kukuczka Academy of Physical Education in Katowice, Katowice, Poland; 3 Division of Clinical Physiotherapy, Faculty of Physical Education, Gdansk University of Physical Education and Sport, Gdańsk, Poland; 4 Department of Sport Games, Faculty of Physical Education and Sport, Charles University in Prague, Prague, Czech Republic; University of Belgrade: Univerzitet u Beogradu, SERBIA

## Abstract

**Purpose:**

Evidence directly comparing the effects of bilateral and unilateral conditioning activities is limited. Therefore, the aim of this study was to assess the acute effect of unilateral and bilateral conditioning activity on vastus lateralis stiffness, countermovement jump parameters, and 10 m sprint.

**Methods:**

Twelve semi-professional basketball players participated in this study (age: 23 ± 4 yrs; body mass: 84.7 ± 10.6 kg; body height: 192 ± 6 cm; basketball training experience: 11 ± 4 yrs) performed four experimental sessions to compare the acute effects of bilateral, stronger-only, weaker-only lower limb or no conditioning activity on vastus lateralis stiffness, countermovement jumps variables (height; peak velocity; peak force, contraction time, countermovement depth, and modified reactive strength index and 10 m sprint time. Measurements were performed 5 minutes before and in the 5^th^ and 10^th^ minutes after CA.

**Results:**

Bilateral conditioning activity significantly increase the countermovement jump height (p = 0.002; ES = 0.71) and the reactive strength index modified (p = 0.010; ES = 0.59). Moreover, a significantly higher peak force in the stronger than in the weaker limb was found (p<0.001) without any differences between conditions and time points (p>0.05). However, there were no significant (p>0.05) interactions and effects of conditions or time-point in the case of the other countermovement jump variables, vastus lateralis stiffness, and 10m sprint time.

**Conclusion:**

Unilateral and bilateral drop jumps (3 sets of 5 repetitions) did not affect the vastus lateralis stiffness and time of the 10m sprint. However, only bilateral drop jumps effectively enhanced the countermovement jump height and modified reactive strength index. Bilateral drop jumps might be a useful part of a warm-up to improve jumping performance in basketball players.

## Introduction

The post-activation performance enhancement (PAPE) effect refers to the temporary improvement in muscular performance following the completion of high-intensity exercise. In practice, the PAPE effect is elicited by the use of a conditioning activity (CA) (i.e., back squat) which precedes a biomechanically similar fast dynamic activity (i.e., vertical jump) [1]. PAPE is particularly useful in sports that require quick force and explosiveness, such as jumping and sprinting, therefore coaches and practitioners aim to effectively induce this phenomenon during training sessions or as part of a pre-competition warm-up [2].

A large number of studies attempted to induce PAPE using bilateral CAs, despite the fact that a majority of motor tasks in sports are performed unilaterally, although the assumption is that the movement pattern of the CA and the subsequent exercise should be comparable [1, 3, 4]. Recent studies have shown that local alterations in the exercising muscle, like elevated temperature and fluid shifts, which affect muscle metabolism and contraction mechanics, maybe the origin of PAPE. Therefore, the CAs utilized as a part of a pre-competition or pre-training warm-up should target the same muscle group that will be engaged in the upcoming exercise, ideally unilaterally, in order to better replicate the subsequent athletic performance. For example, Dello Iacono et al. [5] evaluated countermovement jump (CMJ), 10 m sprint, and change of direction performance after both vertical and horizontal alternating single-leg drop jumps (3 sets of 5 repetitions on each leg, from 25 cm). The findings showed that CA had a force vector-specific impact, with vertical drop jumps improving CMJ performance while horizontal drop jumps increased the change of direction test time. However, the authors did not directly compare those CAs with their bilateral counterparts, thus it is unknown whether unilateral CA might be superior. On the other hand, one of the few studies that directly compared unilateral and bilateral CAs [6] demonstrated a significant decrease in CMJ height after a split and bilateral squat performed with a load that allowed for a mean propulsive velocity of 0.59 m/s, or approximately 87% 1RM. Hence, there is a lack of solid information regarding the interaction between unilateral and bilateral PAPE effects so as to reach firm conclusions.

The mentioned non-localized manner of the PAPE effect might be seen as a benefit and a way to strategically and individually tailor potentiation techniques. For instance, practicing CA just on a weaker limb could be a way to significantly minimize the inter-limb strength asymmetries during following athletic tasks. Strength asymmetries would seem to have a harmful impact on sport performance tasks, such as change of direction and sport-specific abilities and jumping. Therefore, attempting to acutely reduce inter-limb asymmetries might be advantageous for improving athletic performance. In this case, the CAs may only be carried out on the limb that is weaker in order to significantly reduce asymmetry during the following task. However, to the best of the authors’ knowledge, no study to date investigated how unilateral CA affects inter-limb asymmetry.

The highest PAPE effect tends to occur 5–7 min after low-volume CA (1–3 sets and low repetition range ≤5) utilizing high-intensity exercises (>85% one-repetition maximum [1RM] or plyometric). The disadvantages of unilateral exercises, on the other hand, include higher balancing demands than those of bilateral movements, and as a consequence might induce a higher degree of fatigue. Due to this, employing unilateral CAs may not be the best approach when following the current guidelines for potentiation complexes. Notably, to the best of the authors’ knowledge, only three investigations [6–8] directly compared the effects of bilateral and unilateral CA. Moreover, probably no study reported potential changes in each limb’s involvement in bilateral activities carried out after unilateral CA. Therefore, the aim of this study was to directly compare unilateral and bilateral CA on subsequent CMJ performance and 10m linear sprint time with simultaneous evaluation of symmetry index and vastus lateralis stiffness as a muscle fatigue indicator. It was hypothesized that bilateral and unilateral CA performed by the weaker limb will contribute to a significant improvement in selected tests, while mentioned unilateral CA will reduce the symmetry index.

## Materials and methods

### Experimental approach to the problem

**Fig 1:**
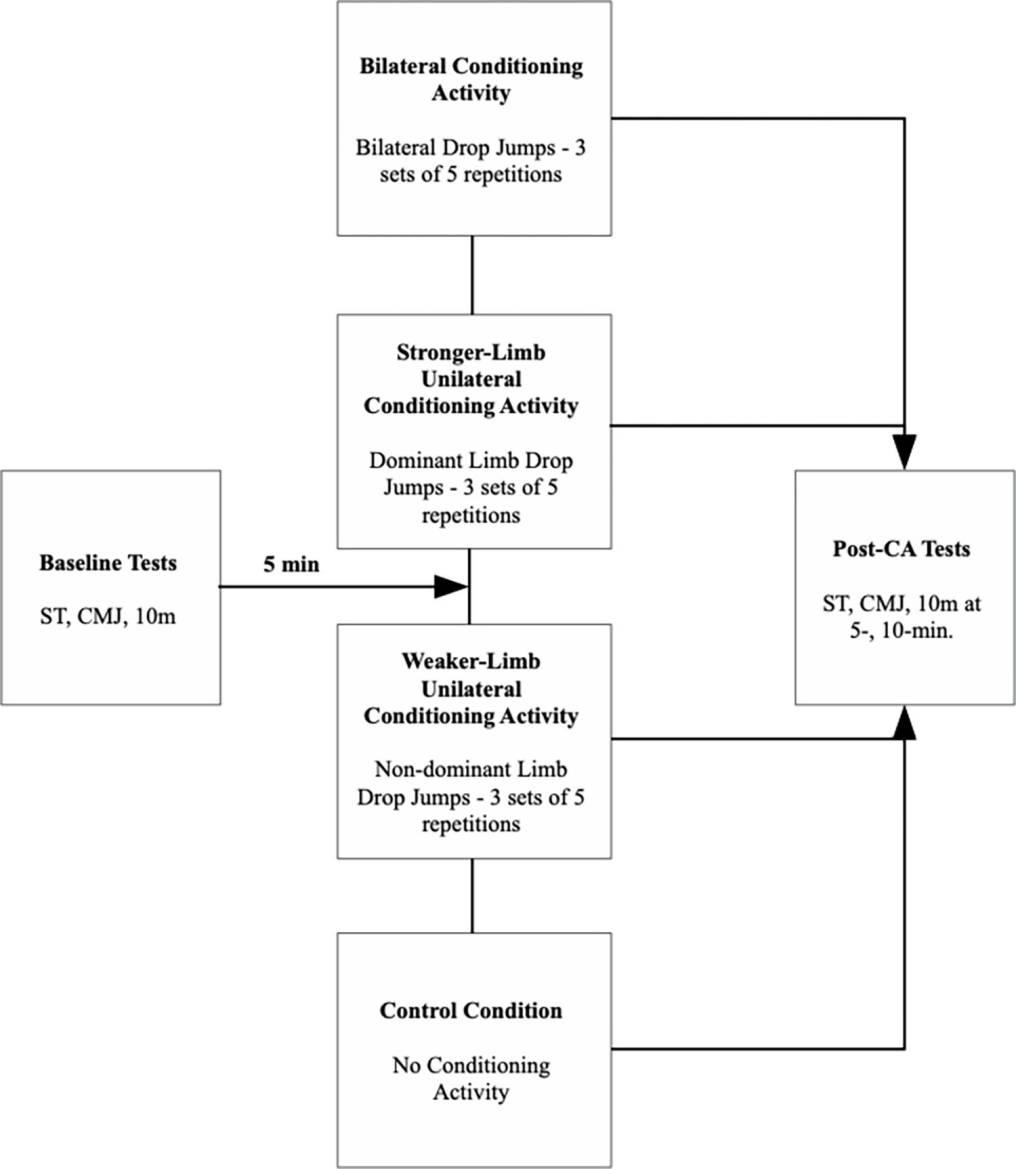
Study design. ST–vastus lateralis stiffness assessment; CMJ–countermovement jump; 10 m– 10 m sprint time; CA- conditioning activity.

The experiment was performed following a randomized crossover design, where each participant performed four experimental sessions to compare the acute effects of bilateral (BI-CA), stronger-only (S-CA), weaker-only (W-CA) lower limb or no CA (CTRL) on vastus lateralis stiffness, countermovement jumps selected kinematics variables and 10 m sprint time. Measurements were performed 5 minutes before and in the 5^th^ and 10^th^ minutes after CA (Fig 1).

### Participants

Twelve semi-professional basketball players participated in this study (age: 23 ± 4 yrs; body mass: 84.7 ± 10.6 kg; body height: 192 ± 6 cm; basketball training experience: 11 ± 4 yrs). The inclusion criteria were as follows: i) free from neuromuscular and musculoskeletal disorders, ii) no lower-limb surgery for two years prior to the study, iii) have at least two years’ experience in basketball training and competition iv) take part in regular basketball and resistance training for at least year prior to the study. Participants were instructed to maintain their usual dietary and sleep habits, and not to use any stimulants and alcoholic drinks throughout the study. Moreover, they were asked not to perform any additional resistance exercises 48-h before testing to avoid fatigue. The study was conducted in February and March 2022. Participants were allowed to withdraw from the experiment at any time and were informed about the benefits and potential risks of the study before providing their written informed consent for participation. Participants were known to the authors, which made it possible to recognize them. Participants were not told of the expected study outcomes. The study protocol was approved by the Bioethics Committee for Scientific Research, at the Academy of Physical Education in Katowice, Poland (3/2021) and performed according to the ethical standards of the Declaration of Helsinki 2013. The sample size was calculated a priori based on a statistical power of 0.8, an effect size of g = 0.37–0.72, and a significance level of 0.05, taking acute changes in stiffness after exercise, and post-activation performance enhancement in jumping performance [9, 10] as a reference variable. A minimum sample size of between 8–12 individuals was obtained (G*Power [version 3.1.9.2], Dusseldorf, Germany).

### Procedures

#### Familiarization session

To avoid the influence of circadian rhythm on performance and maintain the usual training routine, all study sessions were performed between 17:00 p.m. and 19:00 p.m. Three days before the first experimental session a test of the optimal drop jump box height (based on the highest reactive strength index determined using a force platform) and stronger limb determination (based on higher peak force reached) was carried out. After a warm-up consisting of 5 minutes of jogging followed by 8 minutes of dynamic activities and 4 practice countermovement jump as described in detail here [2]. All participants made two maximum jumps from 2 heights for a single leg (30, and 40 cm wooden box) and 2 for a bilateral drop jump (60 and 76 cm wooden box). This initiate the drop action, the participants were instructed to: “step off” the box one foot at a time (left limb first) and then to “jump up as fast as possible after contact with the ground, making sure that the jump is the highest possible”. This instruction aimed to prevent from jumping out of the box. The participant was instructed to perform the contact phase and landing phase on the force plate. The jump was invalid if the participant raised the feet during the jump flight, landed behind the force plate, or jumped off the box during the drop jump.

Moreover, all participants performed 2 attempts of the 10m sprint to familiarize themselves with the study protocol.

#### Experimental session

After the warm-up and baseline assessments (vastus lateralis stiffness, countermovement jump, and 10 m sprint time), the participants performed bilateral, unilateral CA, or no CA in a randomized order. The CA consisted of 3 sets of 5 bilateral drop jumps as a bilateral CA (B-CA) or 3 sets of 5 unilateral drop jumps as a stronger or weaker CA (S-CA or W-CA); depending on which limb it was performed). In the CTRL condition, no CA was applied. Following that, approximately at the 5^th^ and 10^th^ minutes after CA, all measurements were re-tested in the same order. The experimental sessions were performed at least 3 days apart but no more than 5 days. A 2 min rest interval was permitted between sets of drop jumps. The mean of the relative peak force was registered to compare landing forces between conditioning activities.

#### Measurement of countermovement jump performance

The countermovement jump performance was measured using force plates (Force Decks, Vald Performance, Australia). This device has been previously confirmed as a valid and reliable [11] for assessing vertical jump kinematics. Each participant performed two CMJs with hands placed on the hip for each lower limb at pre-CA as a baseline and in the 5^th^ and 10^th^ minutes post-CA. The participants were instructed to perform a quick downward movement at a self-selected depth and, afterward, a fast-upward movement to jump as high as possible. The jump height, peak velocity, RSImod, contraction time, countermovement depth, and peak force were evaluated. The best of the two attempts in terms of jump height was included for analysis. The impulse-momentum theorem was used to calculate jump height. The peak force produced by both limbs was used to calculate a symmetry index score using the following equation [12]:

SI=(stronger−weaker)÷(stronger+weaker)×100

#### Measurement of 10m sprint time

The athletes were instructed to sprint as quickly as possible linearly from the starting point for 10 m. Sprint times were recorded using timing photocells (SmartSpeed Pro, Fusion Sport, Coopers Plains, Australia), with gates at 0 and 10 m. The height was set at approximately 1 m off the ground, corresponding to participants’ hip height to avoid the timing gates being triggered prematurely by a swinging arm or leg. The participants started with a front foot placed 0.3 m behind the first timing gate to prevent any early triggering of the photocells. Times were measured to the nearest 0.001 seconds. Two attempts were performed, and the best time was retained for further analysis.

#### Measurement of vastus lateralis muscle stiffness

The MyotonPRO, hand-held myometer (MyotonPRO, Myoton AS, Tallinn, Estonia) was used for non-invasive assessment of vastus lateralis muscle stiffness. This device has previously demonstrated its reliability in assessing the stiffness of the vastest lateralis [13]. The measurement for vastus lateralis was at 50% of the straight-line distance between the greater trochanter and fibulae capitulum [14]. The Myoton’s accelerometer was set at 3200 Hz with an average value obtained from five consecutive measurements (0.4 N for 15 ms).

#### Statistical analysis

All data were analyzed using IBM SPSS Statistics for Macintosh, Version 25.0 (IBM Corp., Armonk, N.Y., USA) and were shown as means with standard deviations (±SD) with their 95% confidence intervals (CI). Statistical significance was set at p < 0.05. The normality of data distribution was verified using Shapiro–Wilk tests and Mauchly’s test was used to test the assumption of sphericity. The relative (two-way mixed effects, absolute agreement, single rater intraclass correlation coefficient) and absolute (coefficient of variation) reliability were calculated. The thresholds for interpreting intraclass correlation coefficient results were: <0.5 “poor”, 0.5–0.75 “moderate”, <0.76–0.9 “good”, and >0.90 as “excellent” [15]. While the coefficient of variation results was: <10% “very good”, 10–20% “good”, <21–30% “acceptable”, and >30% “not acceptable” [16]. The two-way ANOVAs (or nonparametric equivalent test) (4 [B-CA; S-CA; W-CA; CTRL] × 2 time-points [pre-CA; post-CA]) were used to investigate the influence of CA on CMJ variables, and sprint time. The three-way ANOVAs (4 [B-CA; S-CA; W-CA; CTRL] × 2 time-points [pre-CA; post-CA] × 2 sides [STR; WEAK]) were used to investigate the influence of CA on peak force during CMJ, and vastus lateralis stiffness. Bonferroni correction was used as a post-hoc. The magnitude of mean differences was expressed with standardized effect size (ES). Thresholds for qualitative descriptors of Hedges g were interpreted as ≤0.20 “small”, 0.21–0.79 “medium”, and >0.80 as “large”.

## Results

The Shapiro-Wilk test did show a statistically significant violation of data distribution only for the asymmetry index. In the case of the main effect of the condition for the 10m sprint and weaker limb peak force, the Greenhouse-Geisser correction has been adopted.

The ICC and CV results are presented in Table 1.

**Table 1:** Intersession reliability of the analyzed variables.

Variable	ICC (95%CI)	CV (SD)
Jump Height	0.97 (0.93–0.99)	3.2% (1.7%)
Peak Velocity	0.88 (0.72–0.96)	2.6% (0.9%)
Contraction Time	0.92 (0.80–0.97)	9.3% (2.8%)
Countermovement Depth	0.88 (0.71–0.96)	10.1% (3.3%)
RSImod	0.95 (0.87–0.98)	9.5% (2.7%)
Stronger Limb Peak Force	0.92 (0.81–0.97)	6.4% (2.9%)
Weaker Limb Peak Force	0.93 (0.82–0.98)	5.9% (2.3%)
Sprint Time	0.76 (0.46–0.93)	2.6% (1.3%)
VL Stronger Limb Stiffness	0.80 (0.50–0.93)	6.9% (3.7%)
VL Weaker Limb Stiffness	0.87 (0.69–0.96)	6.4% (3.5%)

ICC–intraclass correlation coefficient; CV–coefficient of variation; RSImod–reactive strength index modified; SD–standard deviation; VL–vastus lateralis

### Comparison of landing forces during conditioning activities

**Fig 2:**
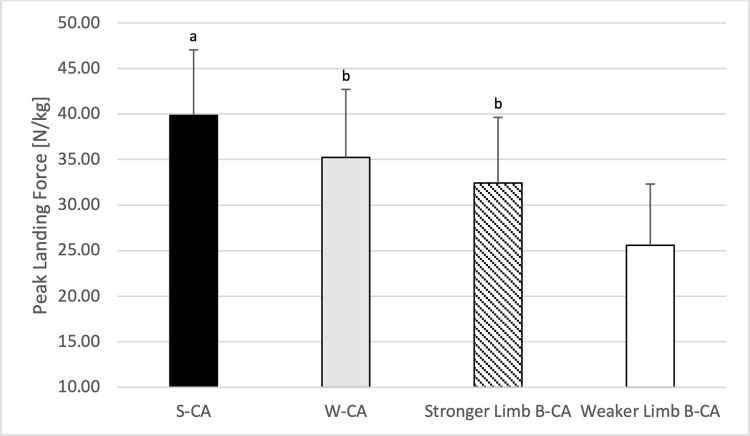
Comparison of peak landing forces during particular conditioning activities. S-CA–stronger limb conditioning activity condition; W-CA–weaker limb conditioning activity condition; B-CA bilateral conditioning activity. a–significant difference in comparison to other conditions; b–significant difference in comparison to the weaker limb during bilateral conditioning activity.

One-way ANOVA showed statistically significant differences between CA in relative landing peak force (F = 24.601; p<0.001; η^2^ = 0.691). The post-hoc analysis indicated a significantly higher relative landing peak force during S-CA in comparison to other CA (p<0.011 for all; ES = 0.58–1.89). Moreover, a higher relative landing peak force during W-CA was found in comparison to the weaker limb during B-CA (p = 0.001; ES = 1.26). Finally, a higher relative landing peak force was found for the stronger limb than for the weaker limb during B-CA (p = 0.035; ES = 0.79) (Fig 2).

### Countermovement jump performance

There was a statistically significant interaction for CMJ height (F = 5.120; p = 0.005; η^2^ = 0.318) and RSImod (F = 3.117; p = 0.039; η^2^ = 0.221). The post-hoc analysis showed a significant increase in CMJ height (p = 0.002; ES = 0.71) and RSImod (p = 0.010; ES = 0.59) post-CA in comparison to pre-CA during the B-CA condition (Table 2).

**Table 2:** Comparison of jumping performance pre- and post-conditioning activity.

Condition	Pre-CA (95%CI)	Post-CA (95%CI)	ES	Percentage Change
Jump Height [cm]				
B-CA	**36.2 ± 3.1 (34.2 to 38.2)**	38.4 ± 2.9* (36.6 to 40.2)	**0.71**	**6.4 ± 5.7%**
S-CA	36.5 ± 4.0 (33.9 to 39.0)	36.8 ± 4.3 (34.1 to 39.5)	0.07	0.9 ± 4.3%
W-CA	36.7 ± 4.0 (34.2 to 39.2)	36.3 ± 4.3 (33.5 to 39.0)	0.09	-1.2 ± 4.6%
CTRL	36.0 ± 3.7 (33.7 to 38.4)	36.4 ± 3.7 (34.0 to 38.7)	0.10	1.0 ± 4.9%
Peak Velocity [m/s]				
B-CA	2.75 ± 0.13 (2.67 to 2.83)	2.82 ± 0.16 (2.73 to 2.92)	0.46	2.7 ± 3.6%
S-CA	2.79 ± 0.12 (2.71 to 2.86)	2.80 ± 0.16 (2.70 to 2.90)	0.07	0.4 ± 2.5%
W-CA	2.80 ± 0.12 (2.72 to 2.87)	2.78 ± 0.19 (2.66 to 2.90)	0.12	-0.5 ± 4.9%
CTRL	2.80 ± 0.14 (2.71 to 2.89)	2.80 ± 0.12 (2.72 to 2.88)	0.00	0.1 ± 3.0%
RSImod				
B-CA	**0.46 ± 0.12 (0.38 to 0.54)**	0.54 ± 0.14* (0.45 to 0.63)	**0.59**	**18.0 ± 18.6%**
S-CA	0.47 ± 0.09 (0.41 to 0.53)	0.50 ± 0.15 (0.40 to 0.59)	0.23	4.7 ± 17.1%
W-CA	0.48 ± 0.12 (0.40 to 0.56)	0.48 ± 0.16 (0.38 to 0.59)	0.00	0.2 ± 11.4%
CTRL	0.47 ± 0.11 (0.41 to 0.54)	0.47 ± 0.11 (0.40 to 0.54)	0.00	-0.4 ± 12.3%
Contraction Time [ms]				
B-CA	815 ± 145 (723 to 907)	744 ± 138 (656 to 832)	0.48	-8 ± 13.6%
S-CA	793 ± 144 (702 to 884)	782 ± 191 (661 to 903)	0.06	-1.6 ± 14.3%
W-CA	797 ± 160 (695 to 899)	796 ± 188 (677 to 916)	0.01	-0.6 ± 7.9%
CTRL	789 ± 145 (697 to 881)	810 ± 185 (692 to 928)	0.12	2.4 ± 10.1%
Countermovement Depth [cm]				
B-CA	26.4 ± 5.2 (23.1 to 29.6)	28.3 ± 5.8 (24.6 to 32.0)	0.33	5.9% ± 16.8%
S-CA	26.4 ± 4.4 (23.6 to 29.2)	26.7 ± 3.1 (24.7 to 28.7)	0.08	1.4% ± 9.4%
W-CA	25.3 ± 4.2 (22.6 to 28.0)	25.9 ± 3.7 (23.6 to 28.3)	0.15	2.2% ± 9.9%
CTRL	26.3 ± 5.3 (22.9 to 29.6)	26.8 ± 6.5 (22.9 to 31.0)	0.08	0% ± 14.1%
Stronger Limb Peak Force [N]				
B-CA	1125 ± 161^#^ (1023 to 1228)	1186 ± 179^#^ (1072 to 1299)	0.35	5.9 ± 11.4%
S-CA	1206 ± 163^#^ (1102 to 1309)	1250 ± 196^#^ (1125 to 1375)	0.24	3.5 ± 3.6%
W-CA	1181 ± 171^#^ (1073 to 1290)	1177 ± 167^#^ (1071 to 1284)	0.02	-0.2 ± 5.5%
CTRL	1184 ± 155^#^ (1085 to 1282)	1193 ± 120^#^ (1116 to 1269)	0.06	1.2 ± 4.7%
Weaker Limb Peak Force [N]				
B-CA	1066 ± 145 (974 to 1158)	1121 ± 157 (1022 to 1221)	0.35	5.6 ± 9.6%
S-CA	1136 ± 141 (1047 to 1226)	1178 ± 162 (1075 to 1281)	0.27	3.6 ± 5.1%
W-CA	1117 ± 135 (1031 to 1202)	1120 ± 132 (1036 to 1204)	0.02	0.4 ± 4.9%
CTRL	1102 ± 138 (1015 to 1189)	1103 ± 144 (1011 to 1195)	0.01	0.1 ± 4.2%

CA–conditioning activity; CI–confidence interval; ES—effect size; B-CA–bilateral conditioning activity condition; S-CA–stronger limb conditioning activity condition; W-CA–weaker limb conditioning activity condition; CTRL–control condition; RSImod–modified reactive strength index; *–a significant difference in comparison to pre in a particular condition; ^#^—a significant difference in comparison to the corresponding time points in weaker limb

However, there were no statistically significant interactions for peak velocity (F = 1.530; p = 0.225; η^2^ = 0.122), contraction time (F = 2.550; p = 0.072; η^2^ = 0.188), countermovement depth (F = 0.490; p = 0.692; η^2^ = 0.043), and peak force (F<1.832; p>0.161; η^2^<0.143). Similarly, there were no main effects of the condition for peak velocity (F = 0.044; p = 0.988; η^2^ = 0.004), contraction time (F = 0.294; p = 0.829; η^2^ = 0.026), countermovement depth (F = 0.846; p = 0.479; η^2^ = 0.071), and peak force (F = 3.296; p = 0.061; η^2^ = 0.231). Moreover, there were no significant main effects of time-point for peak velocity (F = 2.829; p = 0.121; η^2^ = 0.205), contraction time (F = 1.003; p = 0.338; η^2^ = 0.084) and countermovement depth (F = 3.026; p = 0.110; η^2^ = 0.216). However, a significant main effect of the side (F = 44.137; p<0.001; η^2^ = 0.800) showing a higher peak force in the stronger than in the weaker limb was found (Table 2).

**Fig 3:**
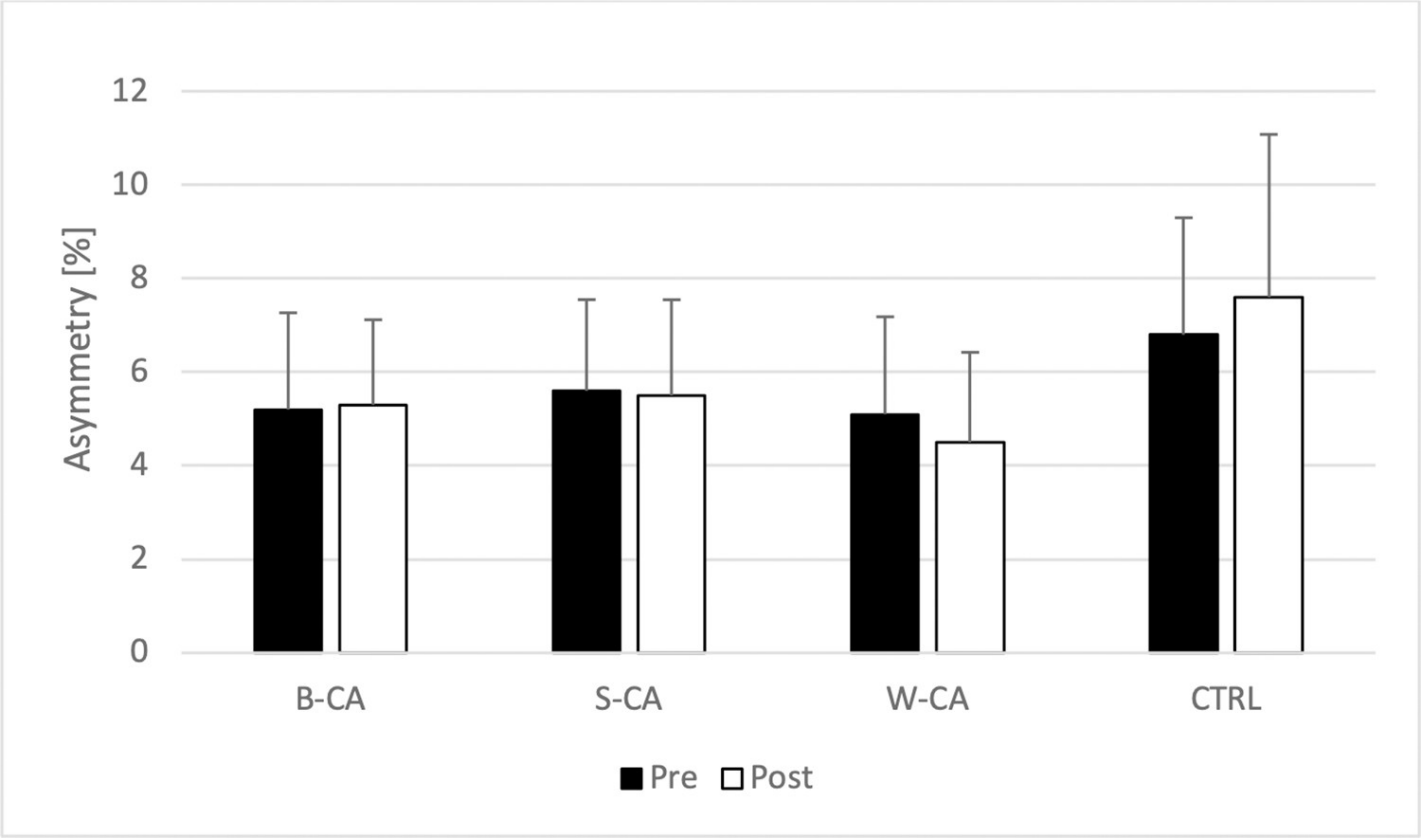
Comparison of inter-limb asymmetry. B-CA–bilateral conditioning activity condition; S-CA–stronger limb conditioning activity condition; W-CA–weaker limb conditioning activity condition; CTRL–control condition.

Friedman’s test didn’t show any differences in inter-limb asymmetries (test = 6.070; p = 0.532; Kendall’s W = 0.72) (Fig 3).

### 10 m sprint time

**Fig 4:**
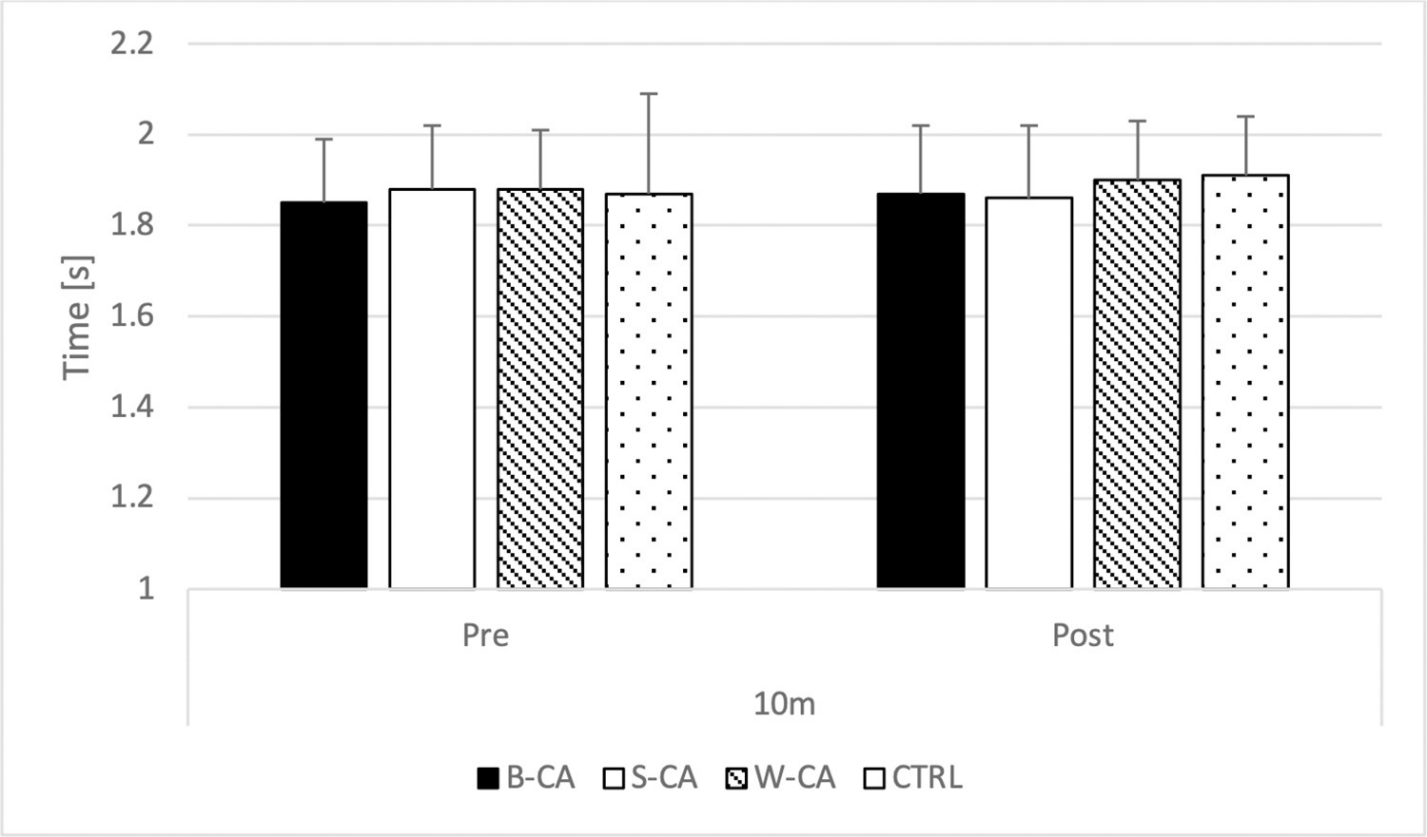
Comparison of 10m sprint time. B-CA–bilateral conditioning activity condition; S-CA–stronger limb conditioning activity condition; W-CA–weaker limb conditioning activity condition; CTRL–control condition.

Two-way ANOVA indicated no statistically significant interaction (F = 1.650; p = 0.197; η^2^ = 0.130), nor the main effect of condition (F = 1.341; p = 0.278; η^2^ = 0.109), and a main effect of time-point (F = 0.628; p = 0.445; η^2^ = 0.054) for 10m sprint time (Fig 4).

### Vastus lateralis stiffness

**Fig 5:**
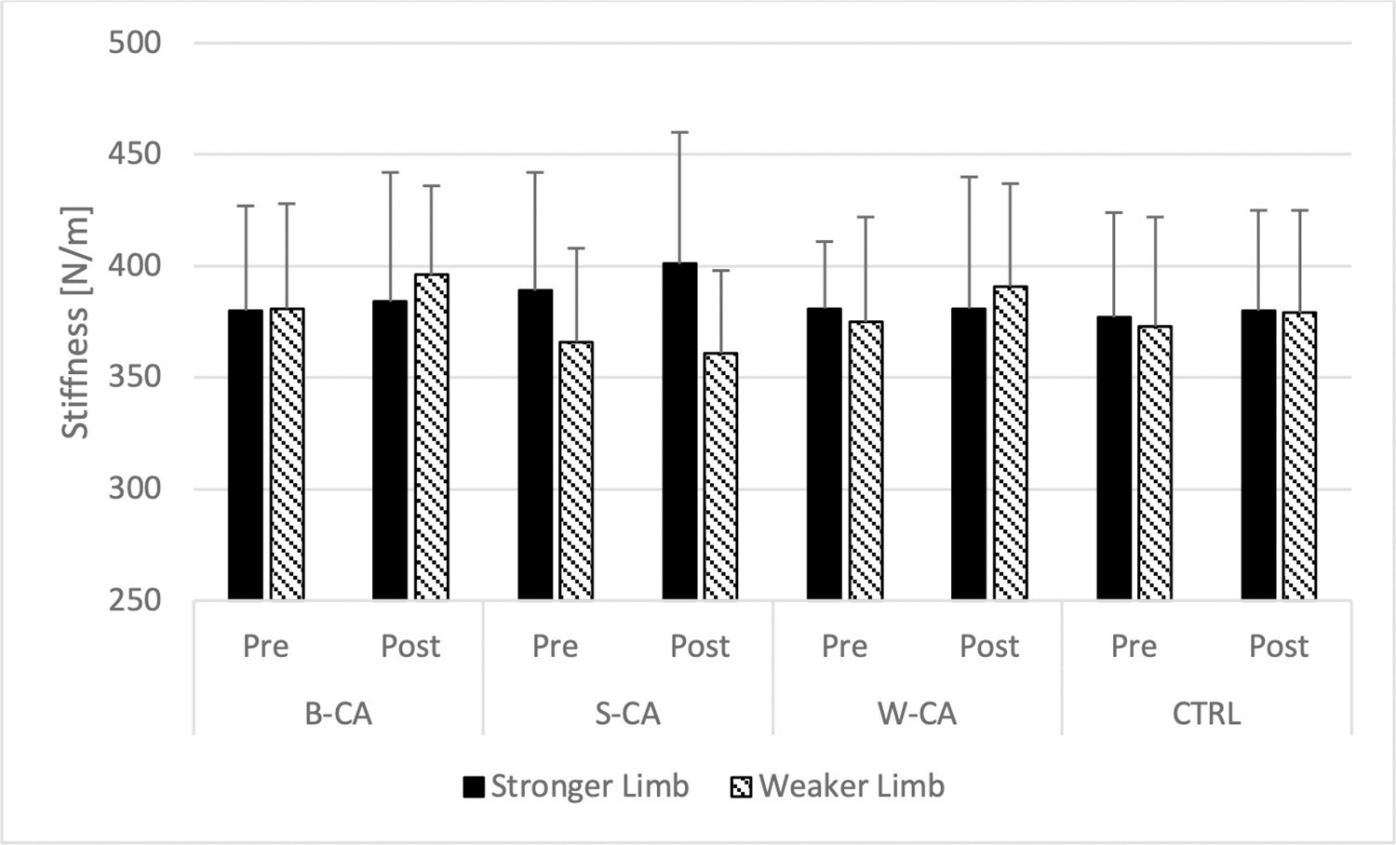
Comparison of vastus lateralis stiffness. B-CA–bilateral conditioning activity condition; S-CA–stronger limb conditioning activity condition; W-CA–weaker limb conditioning activity condition; CTRL–control condition.

Three-way ANOVA didn’t indicate any statistically significant interactions (F<1.979; p>0.136; η^2^<0.152), nor the main effect of condition (F = 0.265; p = 0.850; η^2^ = 0.023) time-point (F = 1.602; p = 0.232; η^2^ = 0.127) and side (F = 0.297; p = 0.597; η^2^ = 0.026) (Fig 5).

## Discussion

The main aim of this study was to compare the effectiveness of bilateral and unilateral drop jumps as a CA on subsequent CMJ performance and sprinting time, as well as inter-limb asymmetry and vastus lateralis stiffness. The main finding of this study was that only B-CA contributed to a significant increase in CMJ height, and RSImod (via a decrease in contraction time). In turn, none of the studied CAs had a significant effect on the 10m sprint time. There were also no significant changes in inter-limb asymmetry or vastus lateralis stiffness during either condition.

To the best of the authors’ knowledge, this is the first study that compared the effects of single-legged and double-legged drop jumps as a CA on subsequent CMJ performance and sprinting time. Although the effectiveness of unilateral exercises in inducing the PAPE effect is well researched, possibly only two studies to date have investigated the use of single-legged drop jumps as a CA. Studies by Dello Iacono et al. [5] showed that single-legged drop jumps contribute to both immediate improvements in CMJ height and change of direction test time in elite handball players. However, it should be emphasized that the participants performed drop jumps in an alternate manner (repetition to repetition), so both limbs were involved, while, to the best of the authors’ knowledge, studies in which CA was "pure unilateral" are limited to the use of isokinetic conditions [17–20]. Furthermore, in none of those studies, performance in the following bilateral task was evaluated.

However, both DJs performed by the stronger and weaker lower limbs used in this study had no effect on the subsequent CMJ performance. On the other hand, according to previous findings, bilateral DJ as a CA increased CMJ height and reduced contraction time, resulting in improved RSImod [21–23]. During unilateral CA, a significantly higher level of peak landing force was noted, which could have been a stronger stimulus than bilateral CA and thus could have caused an unfavorable ratio of potentiation to fatigue. On the other hand, after unilateral CA as well as after bilateral CA, there were no significant differences in the vastus lateralis stiffness, which could indicate induced fatigue [24–26]. However, it should be noted that the ankle joint experiences greater loads during a jump compared to the knee and hip joints [27] therefore, it cannot be ruled out that the assessment of gastrocnemius muscle stiffness would show developing fatigue.

The study included trained athletes, who should have no trouble alternating various exercises and performing them with the highest quality. However, it seems that the obtained results may also be related to the preservation of movement patterns. This may be confirmed by a significant augmentation of the CMJ height with a lack of effects on the 10m sprint time after B-CA. That’s in line with a study by Dello Iacono et al., [5] which showed that 3 sets of 5 repetitions of single-legged DJ performed alternately (in a vertical and horizontal manner) had no significant effect on the 10m sprint time. On the other hand, also in the mentioned study, vertical DJ contributed to a significant improvement in CMJ height, but horizontal ones did not. The authors explained these findings with a similar force-orientation production between vertical DJ and CMJ, hence the lack of enhancement after horizontal DJ. In this study, the force-orientation production in each CA was vertical as in CMJ, however, unlike in the study by Dello Iacono et al., [5] unilateral CAs were performed only by a single limb. Thus, it appears that in PAPE complexes where the post-CA exercise is performed bilaterally, this requires CA involving both limbs, either simultaneously (as in this study) or alternately (as in the Dello Iacono et al. [5]). Nevertheless, the lack of effect due to unilateral CAs might also be related to the stability requirements during these jump types, making the subjects unable to induce the required potentiation level. Moreover, because unilateral motor tasks are more difficult to perform, once an athlete has mastered a movement, it can be difficult to reorganize for the next movement.

Probably none of the previous studies examined the effect of pure unilateral CA on bilateral movement, including measuring the symmetry between the limbs. If the weaker limb performs the CA, the potential local effect of the PAPE suggested in earlier studies [18] could be a way to temporarily reduce the inter-limb strength asymmetry. However, the findings of this study did not confirm that, showing no significant effect on limb asymmetry. Nevertheless, only the level of asymmetry during the bilateral task was assessed in this study. However, unilateral jumps may be a better measure of "actual limb capacity" and therefore potentially more sensitive to detecting asymmetries [28]. Interestingly, although not significant, a similar trend in the increase in peak force of both limbs during CMJ was observed after B-CA and S-CA, with a slightly smaller effect size after the former (0.35 vs. 0.24–0.27). On the one hand, this may to some extent indicate a non-localized effect of PAPE after S-CA since an increase in peak force was also noted in the weaker limb. In turn, this is contradicted by the lack of differences in peak force generated during CMJ after W-CA by both the stronger and weaker limbs. It seems that it is also related to the phenomenon of the maintenance of a motor task and the learning effect. Theoretically, a weaker limb may need more time to adapt and optimally perform a motor task, which may make it more difficult to immediately switch to the next motor task [29].

Several limitations should be taken into account when interpreting the results of this study. First, the protocol of this study did not include a purely unilateral post-CA exercise; therefore, given the similarity of the movement pattern, maybe the applied unilateral DJ would be effective if the post-CA exercise were also purely unilateral. In addition, the CA volume used was the same, even though unilateral CA intensity was significantly greater compared to B-CA, so it is uncertain whether unilateral CA performed at a lower volume would also be ineffective in improving following performance. Moreover, the measurement of stiffness was limited to the measurement of the vastus lateralis only, so it is not known how the other muscle groups, which play a key role in vertical jumping and sprinting, responded. Future studies should consider the use of single-leg DJs of varying volumes, especially a lower number of sets than in this study, and their effect on a purely unilateral post-CA task.

## Conclusions

The results of this study showed that 3 sets of 5 repetitions of single leg DJ performed by both the stronger and the weaker limb did not contribute to a significant improvement in subsequently performed CMJ and 10m sprint as well as did not affect the symmetry between the limbs. On the other hand, the bilateral DJ significantly improved the CMJ jump performance, but not the 10m sprint time in the studied group of basketball players. Coaches and practitioners could use bilateral DJs as the CA during complex training sessions or as part of a pre-competition warm-up to acutely improve jumping performance. However, the individuals should not expect an increase in 10m sprint performance, and changes in vastus lateralis stiffness.
